# Phytochemical Characterization and Effects of *Randia monantha* Benth. Leaf and Fruit Extracts on the Viability of Human Cancer Cell Lines

**DOI:** 10.3390/molecules31173095

**Published:** 2026-09-03

**Authors:** Eugenia Elisa Delgado-Tiburcio, Ivonne Pérez-Bautista, Ramón Marcos Soto-Hernández, Lucero del Mar Ruiz-Posadas, Guadalupe Elizabeth Jimenez-Gutierrez, Axelle Renodon-Cornière, Sara Elisa Herrera-Rodríguez, Ahtziri Socorro Carranza-Aranda, Jonathan J. Magaña, José M. Padrón, Luz A. Guerrero-Lagunes

**Affiliations:** 1 Botany Department, Colegio de Postgraduados, Campus Montecillo, km 36.5 Carretera México-Texcoco, Texcoco 56230, Mexico; delgado.eugenia@colpos.mx (E.E.D.-T.); perez.ivonne@colpos.mx (I.P.-B.); guerrero.luz@colpos.mx (L.A.G.-L.); 2 Genomic Medicine Laboratory, Instituto Nacional de Rehabilitación Luis Guillermo Ibarra Ibarra, Mexico City 14389, Mexico; gjimenezg@cinvestav.mx (G.E.J.-G.); magana.jj@tec.mx (J.J.M.); 3 Nantes Université, Centre National de la Recherche Scientifique (CNRS), Unité en Sciences Biologiques et Biotechnologies (US2B), UMR 6286, F-44000 Nantes, France; axelle.renodon-corniere@univ-nantes.fr; 4 Southeast Unit, Centro de Investigación y Asistencia en Tecnología y Diseño del Estado de Jalisco (CIATEJ), Carretera km 5.5, Sierra Papacal–Chuburná, Chuburná, Merida 97302, Mexico; sherrera@ciatej.mx; 5 Department of Philosophical, Methodological, and Instrumental Disciplines, University Center for Health Sciences (CUCS), University of Guadalajara, Guadalajara 44340, Mexico; ahtziricarranza19@gmail.com; 6 Department of Bioengineering, School of Engineering and Sciences, Tecnologico de Monterrey, Mexico City Campus, Mexico City 14380, Mexico; 7 BioLab, Instituto Universitario de Bio-Orgánica “Antonio González” (IUBO-AG), Universidad de La Laguna, Astrofísico Francisco Sánchez 2, E-38206 La Laguna, Spain; jmpadron@ull.es

**Keywords:** *Randia monantha*, cell viability, secondary metabolites, HPLC

## Abstract

*Randia monantha*, commonly known as crucetillo, is a medicinal plant traditionally used in Mexico to treat conditions such as diabetes, venomous animal bites, and cancer; however, its effects on cancer cell viability remain underexplored. In this study, hexane, ethyl acetate, and methanolic extracts from the leaves and fruits of *Randia monantha* were phytochemically characterized and evaluated for their effects on cell viability in HeLa cervical cancer and DU-145 prostate cancer cell lines using the MTT assay. Phytochemical analyses revealed differences in metabolite composition across solvents of varying polarity and plant tissues. Methanolic leaf extracts contained higher levels of terpenoids and polyphenols and produced the greatest reduction in HeLa cell viability, with an estimated IC_50_ of 4.87 µg/mL after 48 h of treatment. In contrast, DU-145 cells exhibited a different response pattern depending on the extract evaluated. Correlation analysis suggested that flavonoids were associated with reduced HeLa cell viability. Overall, leaf extracts produced greater reductions in cancer cell viability than fruit extracts, highlighting leaves as a promising but comparatively underexplored source of bioactive compounds in *Randia monantha*.

## 1. Introduction

Cancer is one of the leading causes of death worldwide and represents a major clinical and economic burden [1]. In 2022, the International Agency for Research on Cancer (IARC) estimated approximately 20 million new cancer cases and 9.7 million cancer-related deaths worldwide, highlighting the urgent need for more effective and accessible therapeutic strategies [2]. In this context, natural products have emerged as an important source of bioactive compounds for cancer prevention and treatment, since many currently used anticancer agents are derived from natural molecules [3].

Mexico is recognized as a megadiverse country, harboring approximately 23,424 vascular plant species, of which around 4500 are considered medicinal plants [4]. A significant proportion of the population relies on natural products as accessible alternatives for primary healthcare, partly due to limited access to public health services, which covers only 52.6% of the population [5]. Consequently, traditional knowledge from indigenous and rural communities has played an essential role in identifying and using these species for medicinal purposes [6]. Among them, the genus *Randia* (Rubiaceae), commonly known as “crucetillo,” comprises 64 species distributed in Mexico, including 47 endemic species and at least 19 with ethnobotanical relevance [7,8]. Fruits of the *Randia* species are traditionally used to treat several ailments, including bites from venomous animals, kidney diseases, diabetes, arthritis, headache, hypertension, cancer, and, more recently, COVID-19 [7,9]. Previous studies have also reported antioxidant, antimutagenic [10,11,12], diuretic, urolithiatic [13], anti-snake-venom effects [9,14], and antiproliferative effects associated with this genus [15].

Compounds such as flavonoids, phenolic acids, and terpenoids have been associated with antioxidant and antiproliferative activities across various cancer models, suggesting that phytochemical diversity may inform the development of novel therapeutic approaches. In particular, previous studies on *Randia monantha* have demonstrated that fruit extracts exhibit high antioxidant activity and do not produce toxic effects in murine models over a broad dose range [16]. Likewise, low cytotoxicity of *Randia* spp. fruit extracts have been reported to be safe in J774.2 murine macrophages, suggesting a potentially favorable safety profile for this genus [15]. According to the ethnobotanical review by Torres-Montúfar and Jiménez-Noriega (2025) [7], medicinal use is the most frequently reported category in the genus *Randia*, with fruits being the most commonly used plant part. Nevertheless, information regarding the phytochemical composition and antiproliferative potential of *Randia* spp. against cancer cell lines remains limited. In particular, the relationship between secondary metabolites and biological activity among extracts obtained with solvents of different polarities has not been fully investigated. Additionally, comparative analyses between leaves and fruits are still scarce, despite the fact that different plant tissues may accumulate distinct classes of bioactive compounds that could influence their biological effects.

Therefore, the aim of this study was to characterize the phytochemical profile of crude extracts of *Randia monantha* Benth. obtained using hexane, ethyl acetate, and methanol, and to evaluate their effects on the viability of HeLa and DU-145 cancer cell lines, representing cervical and prostate cancer models, respectively, using the MTT assay. This study seeks to expand scientific knowledge of the phytochemical composition and biological activity of *Randia monantha* and to provide a basis for further investigation of its bioactive potential.

## 2. Results

### 2.1. Extraction Yields of Randia monantha Leaf and Fruit Extracts

The dried methanolic fruit extract of *Randia monantha* showed the highest extraction yield, reaching 11.63%, whereas the ethyl acetate and hexane fruit extracts yielded 1.64% and 0.66%, respectively. For leaf extracts, both the methanolic and ethyl acetate fractions exhibited similar yields (2.29%), while the hexane extract showed the lowest yield (1.31%). These results indicate that polar solvents were more effective in recovering extractable compounds from *Randia monantha*, particularly from fruit tissues.

### 2.2. Preliminary Qualitative Phytochemical Profiling of Randia monantha

Preliminary phytochemical profiling of *Randia monantha* extracts from leaves and fruits following standard protocols revealed the presence of several classes of secondary metabolites, including phenols, terpenoids, alkaloids, tannins, flavonoids, and saponins (Table 1).

Terpenoids were the most abundant group, particularly in ethyl acetate extracts of both leaves and fruits, and in the methanolic extract of fruits. Saponins were predominantly detected in the methanolic extract of the fruit, while flavonoids were only detected in this extract. Alkaloids were present at low levels, mainly in the methanolic extracts of leaves and fruits, and in the leaf ethyl acetate extract. Phenolic compounds were detected in all extracts, with higher intensity in methanolic extracts, and tannins were only detected in the ethyl acetate extract of leaves.

The thin-layer chromatography (TLC) analysis revealed distinct chromatographic profiles among the extracts, with Rf values varying with metabolite class and extraction solvent (Table 2). Bands compatible with flavonoids were detected in LH, LEA, LM, and FEA extracts, with Rf values similar to those of the quercetin standard. Likewise, bands corresponding to phenolics, tannins, alkaloids, saponins, and terpenoids were observed in different extracts, supporting the presence of diverse classes of secondary metabolites. The terpenoid fraction exhibited the widest Rf distribution, suggesting a greater structural diversity of compounds within this metabolite group.

On the other hand, differences in chromatographic profiles were observed among the samples, suggesting that metabolite composition depends on both the type of extract and the solvent used.

### 2.3. Identification of Phenolic Acids, Flavonoids and Terpenes by High-Performance Liquid Chromatography in Extracts of Randia monantha

We used HPLC (High-Performance Liquid Chromatography) to analyze and characterize the phenolic acids, flavonoids, and terpenoid compounds in different *Randia monantha* extracts. The chromatographic analysis revealed differences in the phytochemical profiles depending on both solvent polarity and plant tissue. In addition, the relative abundance of several detected compounds varied among extracts (Supplementary Materials).

Total phenolic content varied significantly among extracts, as determined by one-way ANOVA followed by Tukey’s post hoc test (*p* < 0.001). The methanolic leaf extract showed the highest phenolic concentration, significantly exceeding those of the other extracts. Intermediate phenolic levels were observed in the methanolic and ethyl acetate fruit extracts, with no significant differences between them. In contrast, hexanic extracts from both tissues showed low phenolic contents, while no detectable phenolic compounds were observed in the ethyl acetate leaf extract under the analytical conditions used (Table 3).

Consistent with these findings, flavonoid content differed significantly among the extracts (*p* < 0.001). The methanolic leaf extract had the highest flavonoid concentration, significantly exceeding all other extracts (Table 4). In contrast, the remaining extracts exhibited comparatively low flavonoid levels, with several groups showing no significant differences among them according to Tukey’s post hoc test. The hexane fruit extract showed the lowest flavonoid content. These findings suggest that methanol was particularly effective at extracting flavonoids from leaf tissues, highlighting the influence of both solvent polarity and plant tissue on flavonoid recovery.

Terpenoid content differed significantly among extracts (one-way ANOVA, *p* = 0.003). The methanolic fruit extract had the highest total terpenoid content (203.15 mg/g extract), followed by the ethyl acetate leaf extract (188.61 mg/g extract). According to Tukey’s post hoc test, the ethyl acetate leaf extract differed significantly from the methanolic leaf extract and from several of the remaining extracts, whereas the methanolic fruit extract showed greater variability and therefore was not significantly different from most other treatments. In contrast, the methanolic leaf extract presented the lowest terpenoid content (31.48 mg/g extract) (Table 5). These results indicate that terpenoid accumulation depends on both the plant organ and the solvent polarity, with methanolic fruit and ethyl acetate leaf extracts yielding the highest total terpenoid recoveries.

Beyond differences in total terpenoid content, the composition of individual terpenoids also varied among extracts. Oleanolic acid was the predominant compound in the ethyl acetate and methanolic leaf extracts, whereas stigmasterol predominated in all fruit extracts. Ursolic acid was detected only in leaf extracts, whereas β-sitosterol was mainly associated with fruit extracts. These differences indicate that, beyond variations in total terpenoid concentration, leaf and fruit extracts have distinct terpenoid profiles.

### 2.4. Effects of Randia monantha Extracts on HeLa Cell Viability

The reduction in cell viability of *Randia monantha* extracts against HeLa cells varied with plant organ, solvent type, concentration, and exposure time. In general, lower absorbance values indicated reduced cell viability.

**Figure 1:**
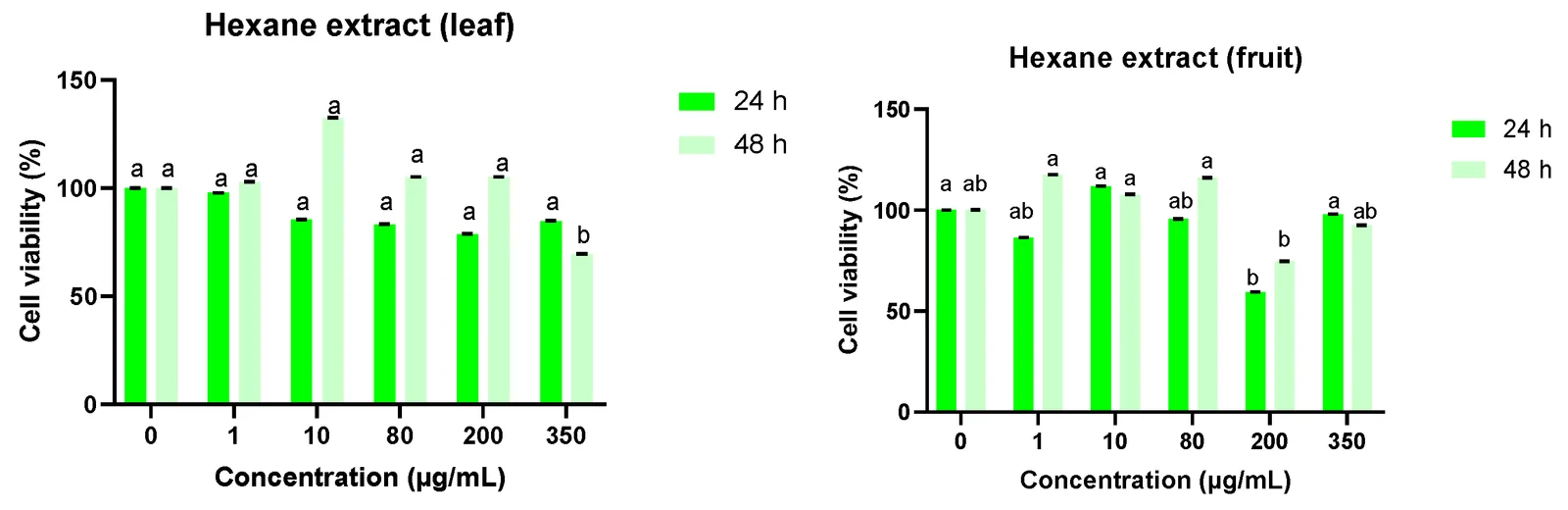
Effect of hexane extracts of *Randia monantha* on HeLa cell viability after 24 h and 48 h of treatment. Cell viability (%) was calculated relative to the untreated cells (C). Error bars represent standard deviation (SD, *n* = 3). Different letters indicate significant differences among treatments according to Tukey’s post hoc test (*p* < 0.05).

For the hexane leaf extract, treatments did not differ significantly at 24 h (*p* > 0.05). In contrast, we detected a significant treatment effect at 48 h (*p* < 0.001). According to Tukey’s post hoc test, only the highest concentration evaluated (350 µg/mL) differed significantly from the control, indicating a modest time-dependent reduction in cell viability. The hexane fruit extract showed significant treatment effects at both 24 h and 48 h (ANOVA, *p* < 0.01). According to Tukey’s post hoc test, the 200 µg/mL treatment exhibited the lowest cell viability and was the only concentration consistently assigned to a distinct statistical group at both time points. Nevertheless, the overall reduction in cell viability remained moderate compared with that observed for the methanolic leaf extract (Figure 1).

**Figure 2:**
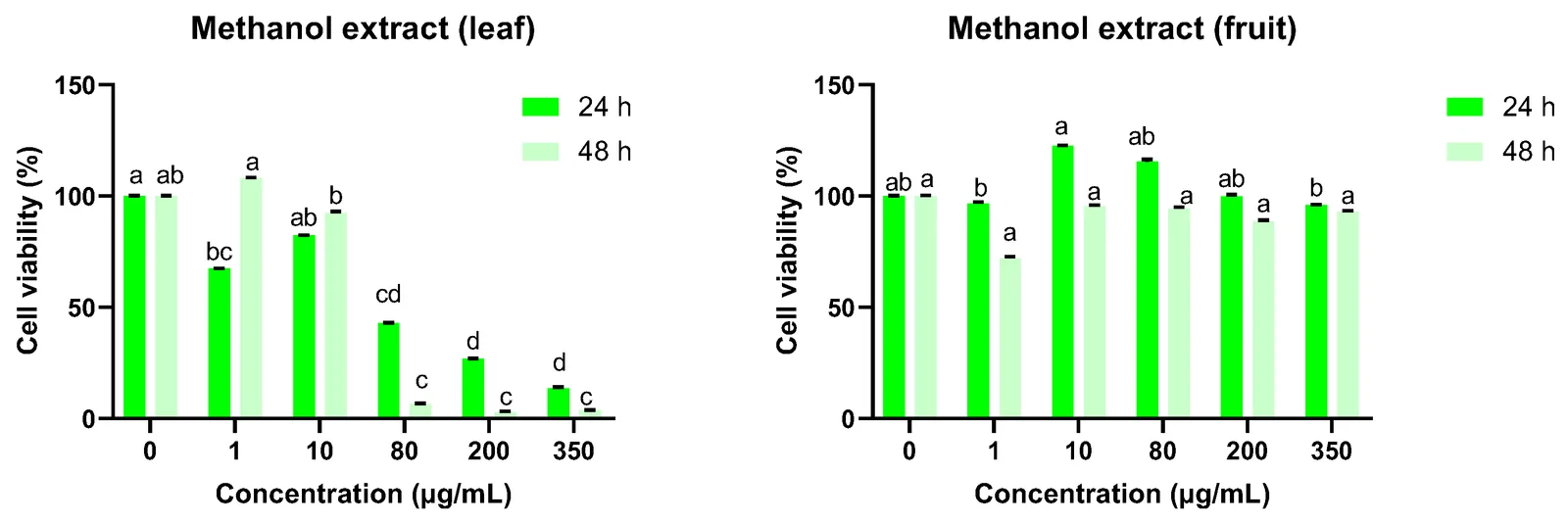
Effect of methanolic extracts of *Randia monantha* on HeLa cell viability after 24 h and 48 h of treatment. Cell viability (%) was calculated relative to the untreated cells (C). Data are presented as mean ± standard deviation (SD) of three independent experiments (*n* = 3). Different letters indicate significant differences among treatments according to Tukey’s post hoc test (*p* < 0.05).

The methanolic leaf extract showed a marked concentration-dependent reduction in cell viability at both 24 h and 48 h; cell viability decreased significantly from 80 µg/mL onward, with the lowest values at 200 and 350 µg/mL. Tukey’s post hoc test showed that the 80, 200, and 350 µg/mL treatments differed significantly from the control at both time points (Figure 2). The IC_50_ values were estimated at approximately 25 µg/mL at 24 h and 4.87 µg/mL at 48 h. These results indicate a greater reduction in cell viability after prolonged exposure. In contrast, the methanolic fruit extract exhibited only minor variations in cell viability across treatments. Although significant treatment effects were detected by ANOVA at both 24 h and 48 h, Tukey’s post hoc test did not reveal significant reductions in cell viability relative to the untreated control. Overall, the methanolic fruit extract showed limited effects on cell viability under the conditions evaluated.

**Figure 3:**
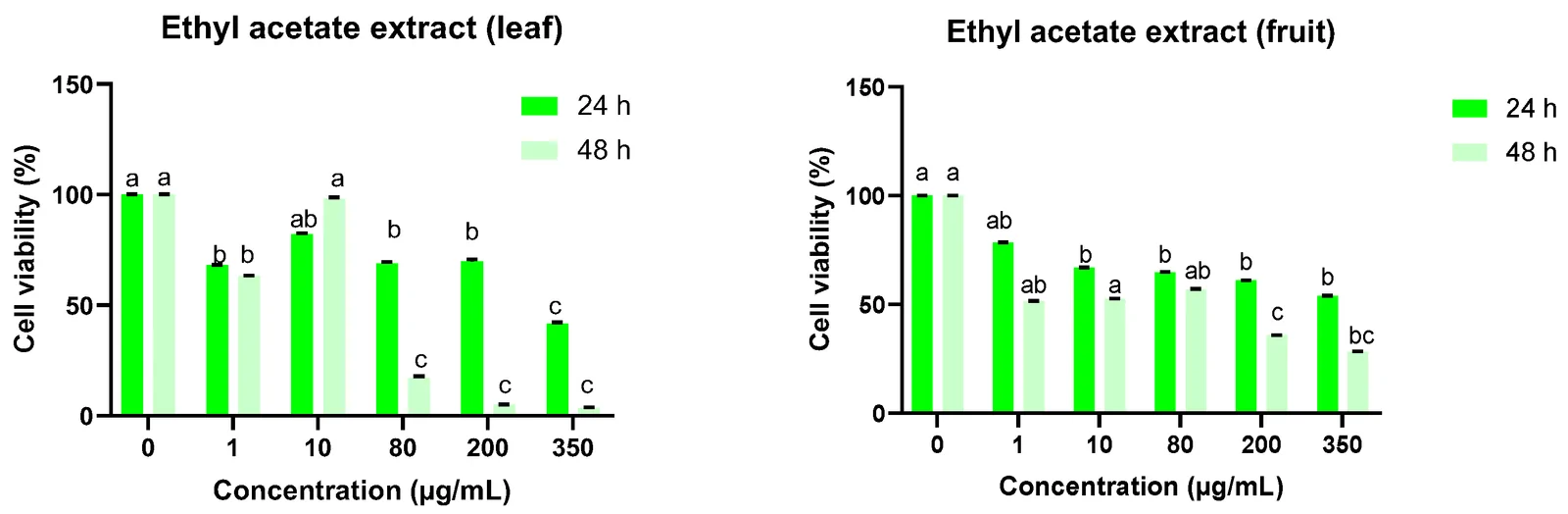
Effect of ethyl acetate extracts of *Randia monantha* on HeLa cell viability after 24 h and 48 h of treatment. Cell viability (%) was calculated relative to the untreated cells (C). Data are presented as mean ± standard deviation (SD) of three independent experiments (*n* = 3). Different letters indicate significant differences among treatments according to Tukey’s post hoc test (*p* < 0.05).

Finally, the ethyl acetate leaf extract produced a marked reduction in cell viability, particularly after 48 h of exposure. Cell viability decreased significantly from 80 µg/mL onward, with the lowest values at 200 and 350 µg/mL. Tukey’s post hoc test showed that the 80, 200, and 350 µg/mL treatments differed significantly from the untreated control at 48 h. The IC_50_ value was approximately 14 µg/mL. In contrast, the ethyl acetate fruit extract produced a moderate reduction in cell viability. At 24 h, treatments of 10 µg/mL and higher showed significantly lower cell viability than the control, according to Tukey’s post hoc test. At 48 h, only the 200 µg/mL treatment differed significantly from the untreated control (Tukey’s post hoc test), whereas the remaining concentrations showed intermediate responses. Although 350 µg/mL showed the lowest mean viability (Figure 3), it was not assigned to a distinct statistical group. Overall, the reduction cell viability of the fruit extract was less pronounced than that observed for the corresponding leaf extract, suggesting a higher abundance or greater effectiveness of bioactive compounds in leaf tissues.

### 2.5. Effects of Randia monantha Extracts on DU-145 Cell Viability

The effects of *Randia monantha* extracts against DU-145 cells was evaluated at 48 h across different solvents and plant tissues, revealing distinct patterns based on extract polarity and origin. Overall, the biological response varied notably among hexane, ethyl acetate, and methanolic fractions, highlighting the influence of solvent extraction on the enrichment of bioactive compounds.

**Figure 4:**
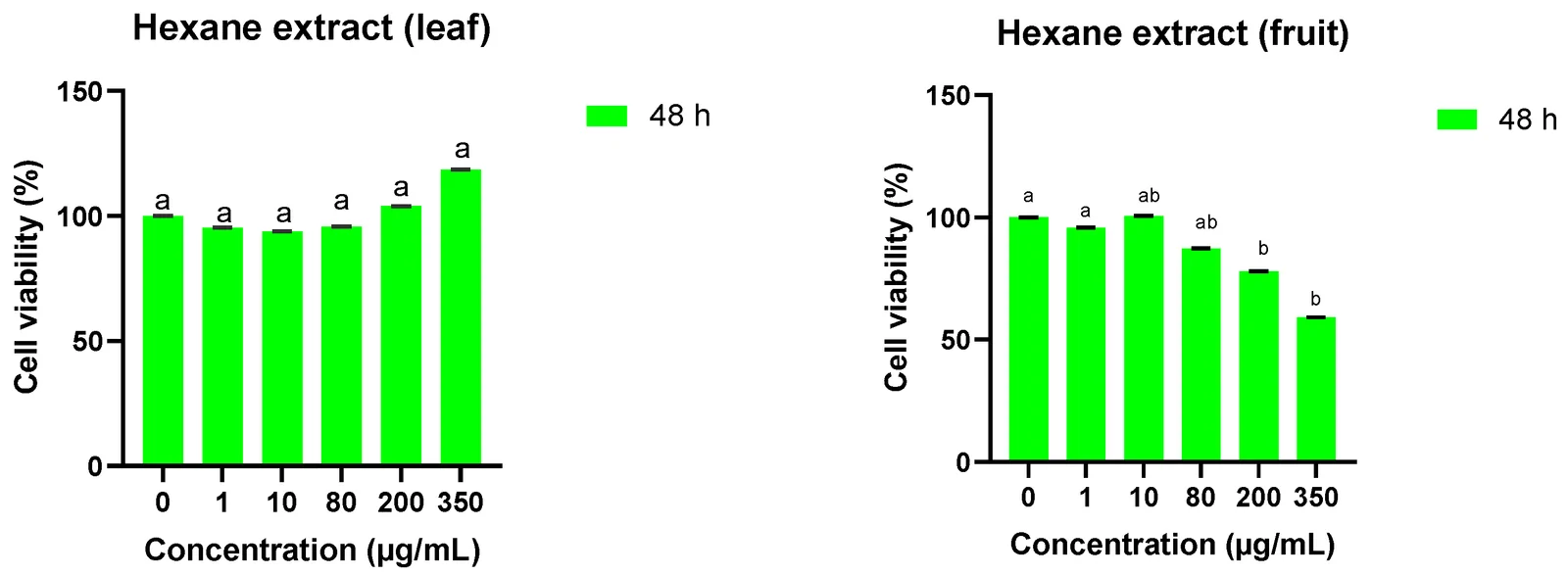
Effect of hexane extracts of *Randia monantha* on DU-145 cell viability after 48 h of treatment. Cell viability (%) was calculated relative to the untreated cells (C). Data are presented as mean ± standard deviation (SD) of three independent experiments (*n* = 3). Different letters indicate significant differences among treatments according to Tukey’s post hoc test (*p* < 0.05).

The hexane extracts exhibited contrasting biological activity across plant tissues. The hexane leaf extract did not show a statistically significant reduction cell viability on DU-145 cells. Although ANOVA indicated no significant treatment effect (*p* = 0.13), the normality assumption was not met; therefore, we also performed a Kruskal–Wallis analysis. This test confirmed the absence of significant differences among treatments (*p* = 0.116), and Dunn’s post hoc test with Bonferroni correction did not reveal significant differences between the control and any extract concentration. We observed no clear dose–response relationship. The hexane fruit extract exhibited a significant treatment effect on DU-145 cells (Kruskal–Wallis, *p* = 0.00024). Cell viability generally decreased with increasing extract concentration, suggesting a dose-related reduction in cell viability. Dunn’s post hoc test with Bonferroni correction showed that the 200 µg/mL treatment significantly reduced cell viability compared with the control (*p* = 0.015), whereas the 350 µg/mL treatment showed a similar reduction but was not statistically significant after correction (*p* = 0.066). No significant differences were detected between the control and the 1, 10, or 80 µg/mL treatments. Overall, these results indicate moderate effect on DU-145 cell viability of the hexane fruit extract against DU-145 cells (Figure 4).

**Figure 5:**
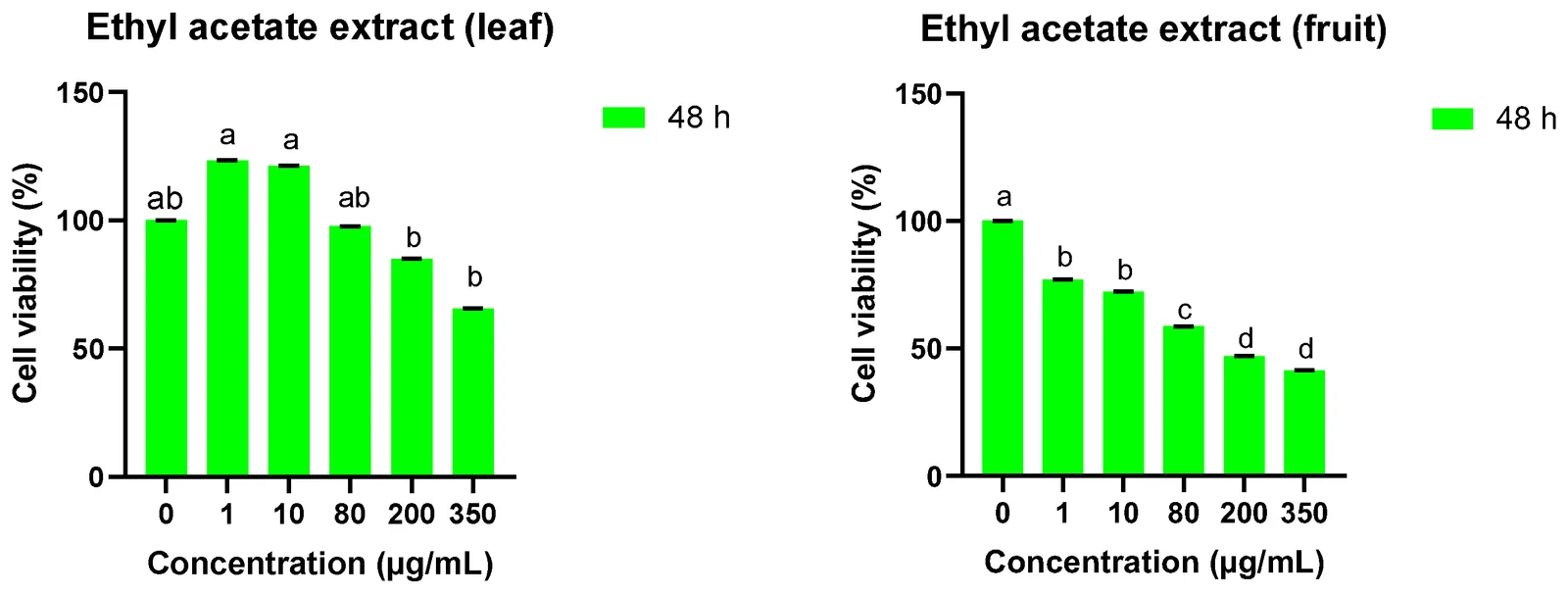
Effect of ethyl acetate extracts of *Randia monantha* on DU-145 cell viability after 48 h of treatment. Cell viability (%) was calculated relative to the untreated control cells (C). Data are presented as mean ± standard deviation (SD) of three independent experiments (*n* = 3). Different letters indicate significant differences among treatments according to Tukey’s post hoc test (*p* < 0.05).

Among all tested fractions, the ethyl acetate extracts produced the greatest reductions in DU-145 cell viability; the ethyl acetate leaf extract showed one of the strongest effects. The Kruskal–Wallis test detected a significant treatment effect (*p* = 0.0005), and cell viability generally decreased as extract concentration increased. Post hoc comparisons indicated that the highest concentrations (200 and 350 µg/mL) were associated with lower cell viability than the low-concentration treatments (1 and 10 µg/mL). However, after Bonferroni correction, no significant differences were detected between the control and any treatment concentration. The lowest mean viability was observed at 350 µg/mL (64.8%), supporting a concentration-dependent trend in the biological response. Similarly, the ethyl acetate fruit extract produced a pronounced reduction in DU-145 cell viability. A highly significant treatment effect was detected (ANOVA, *p* < 0.0001), and cell viability decreased progressively as extract concentration increased. Tukey’s post hoc test showed that concentrations of 80 µg/mL and above had significantly lower viability than the control, with the strongest reductions at 200 and 350 µg/mL. Mean cell viability decreased from 100% in the control to 58.64%, 46.97%, and 41.31% at 80, 200, and 350 µg/mL, respectively. These results indicate a clear concentration-dependent reduction in DU-145 cell viability and suggest that the ethyl acetate fraction may contain bioactive constituents associated with the observed effect (Figure 5).

**Figure 6:**
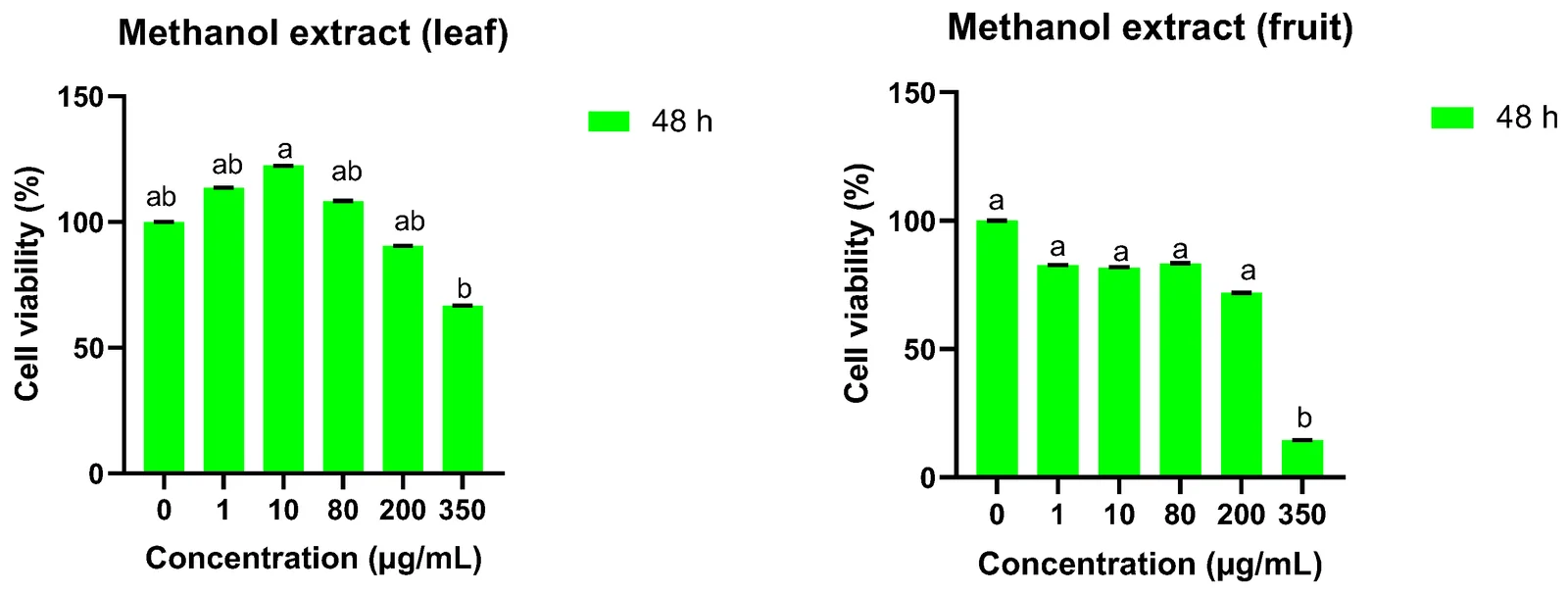
Effect of methanol extracts of *Randia monantha* on DU-145 cell viability after 48 h of treatment. Cell viability (%) was calculated relative to the untreated control cells (C). Data are presented as mean ± standard deviation (SD) of three independent experiments (*n* = 3). Different letters indicate significant differences among treatments according to Tukey’s post hoc test (*p* < 0.05).

Following this trend, the methanolic extracts also exhibited biological activity against DU-145 cells, although the response was less pronounced than that observed for the ethyl acetate extracts. A significant overall treatment effect was detected (Kruskal–Wallis, *p* = 0.034). Cell viability tended to decrease with increasing concentration, reaching its lowest value at 350 µg/mL (66.70%). However, Dunn’s post hoc test with Bonferroni correction did not reveal significant differences between the control and any treatment concentration. The only significant pairwise difference was observed between the 10 and 350 µg/mL treatments. These results suggest a tendency toward reduced cell viability at higher concentrations, although the effect was moderate and less consistent than that observed for the ethyl acetate fraction. In contrast, the methanolic fruit extract displayed a distinct response pattern. We detected a significant treatment effect (Kruskal–Wallis, *p* < 0.001). However, Dunn’s post hoc test with Bonferroni correction revealed that only the highest concentration evaluated (350 µg/mL) significantly reduced cell viability compared with the control. Cell viability decreased from 100% in the control to 14.5% at 350 µg/mL, whereas the lower concentrations (1–200 µg/mL) showed no significant differences. These results indicate a threshold-dependent rather than a gradual dose-dependent response, a substantial reduction in cell viability was observed only at the highest concentration tested (Figure 6).

The IC_50_ was estimated only for the ethyl acetate fruit extract, which showed a clear concentration-dependent decrease in DU-145 cell viability and reached below 50% within the tested concentration range. Using log-linear interpolation, the IC_50_ was estimated at 159 µg/mL. For the remaining extracts, we did not determine IC_50_ values because cell viability did not drop below 50% or because the response did not follow a consistent dose-dependent pattern.

### 2.6. Correlation Between Secondary Metabolite Content and Cell Viability

To further explore the relationship between phytochemical composition and cell viability, Spearman correlation analyses were conducted between the total contents of terpenoids, flavonoids, and phenolic compounds and cell viability at 350 µg/mL, the concentration at which the greatest reductions in cell viability were observed (Table 6).

At 24 h, flavonoid content showed the strongest association with cell viability in HeLa cells, with a significant negative correlation (ρ = −0.829, *p* = 0.042). This result indicates that extracts richer in flavonoids tended to produce greater reductions in cell viability. At 48 h, flavonoids maintained a moderate negative correlation with cell viability (ρ = −0.657, *p* = 0.156), although the association was weaker and no longer statistically significant.

DU-145 cells showed a different pattern. At 48 h, terpenoid content showed a moderate negative correlation with cell viability (ρ = −0.600, *p* = 0.208), whereas flavonoid content showed a relatively strong positive correlation (ρ = 0.771, *p* = 0.072). Phenolic compounds showed only a weak negative association (ρ = −0.143, *p* = 0.787). Although none of these correlations were statistically significant, the contrasting trends between HeLa and DU-145 cells suggest that the metabolite classes associated with changes in cell viability may differ between cell lines.

Because plant extracts contain complex mixtures of bioactive compounds, interpret these results cautiously. Furthermore, the limited number of extracts analyzed (*n* = 6) limits the analysis’s statistical power. Therefore, the observed correlations should be considered exploratory associations within the evaluated extract panel and should not be interpreted as evidence of causality. The observed changes in cell viability may reflect the contribution of multiple bioactive constituents and potential interactions among metabolites rather than the effect of a single phytochemical class.

## 3. Discussion

The present study showed that the phytochemical composition and effects of *Randia monantha* extracts on cancer cell viability were strongly dependent on both the extraction solvent and plant tissue, with leaf extracts, particularly the methanolic and ethyl acetate fractions, producing the greatest reductions in HeLa cell viability.

The extraction yield also varied with solvent polarity and plant tissue. The methanolic fruit extract showed the highest crude extract yield, whereas hexane extracts yielded less. Similar observations have been reported in other medicinal plants, where extraction efficiency strongly depends on solvent polarity and the chemical nature of phytochemicals [17]. However, despite the higher yield in the methanolic fruit extract, the greatest reductions in cell viability were observed primarily in leaf extracts. This suggests that extraction yield alone does not necessarily predict biological activity, since different solvents may selectively extract metabolites with distinct biological properties.

These differences in biological activity were generally consistent with the phytochemical profiles obtained by TLC and HPLC analyses. Preliminary colorimetric phytochemical screening suggested a terpenoid-rich composition in several extracts, whereas TLC and HPLC provided a more detailed characterization of the metabolite composition. Both chromatographic techniques detected flavonoid-related compounds, including in the methanolic leaf extract. However, the preliminary colorimetric screening did not detect flavonoids in this extract. This discrepancy likely reflects the differing analytical capabilities of the methods. Colorimetric phytochemical tests are rapid, qualitative assays with limited sensitivity and specificity, whereas chromatographic techniques such as TLC and, especially, HPLC provide greater sensitivity and resolution, enabling detection of metabolites at low concentrations or in complex mixtures [18].

Previous phytochemical studies have reported several terpenoid compounds in different *Randia* species. *R. hebecarpa* leaves contain triterpenoid glycosides such as cincholic acid 3-O-β-D-quinovopyranosyl-28-O-β-D-glucopyranoside and quinovic acid 3-O-β-D-quinovopyranosyl-28-O-β-D-glucopyranoside. In the ethyl acetate fraction of *R. echinocarpa* fruits, quinovic acid and oxoquinovic acid are abundant constituents, whereas *R. aculeata* leaves contain the diterpene phytol and the triterpenes squalene and cycloartenol [19]. In the present study, oleanolic acid was among the major terpenoids identified, particularly in leaf extracts, and has been widely studied for its anticancer properties [20,21,22]. It has been shown to exert anti-inflammatory effects and inhibit cell proliferation by inducing cell-cycle arrest in various cancer cell lines, including those from prostate and gallbladder cancers [23,24,25]. Oleanolic acid has also shown anti-inflammatory activity in cellular models, including inhibition of HMGB1-mediated inflammatory responses through reduced NF-κB activation and TNF-α production [26]. Its anticancer effects have been demonstrated across a wide variety of cancers, including liver cancer. In a study by Xu et al. [27], oleanolic acid was shown to exert hepatoprotective effects, partly through induction of HMOX1 expression, an antioxidant enzyme that regulates oxidative stress responses such as hypoxia and inflammation. Transcriptomic analyses further suggested that HMOX1 and PIM1 may contribute to liver regeneration and to oleanolic acid’s protective effects against liver injury. In addition, Zhao et al. [28] reported that oleanolic acid inhibited the proliferation of lung carcinoma cells by inducing cell cycle arrest through the miR-122/CCNG1/MEF2D pathway. In contrast, oleanolic acid arrested the cell cycle in lung cells by inducing the expression of transcription factors that regulate miR-122 [27]. Several synthetic derivatives of oleanolic acid, including CDDO, CDDO-Me, CDDO-Im, and the novel derivative SZC017, have also demonstrated potent anticancer activity in experimental models. For example, SZC017 has been reported to induce apoptosis, autophagy, and G0/G1 cell cycle arrest in breast cancer cells [29]. In addition, oleanolic acid can exert antiproliferative effects in osteosarcoma cells [30], pancreatic cancer cells [31], and gastric cancer cells [32].

In addition to terpenoids, phenolic compounds identified in the extracts may also contribute to the observed reduction in cell viability. The profile identified in this study shows both similarities and differences compared to previous reports on *Randia monantha* by Juárez-Trujillo et al. [16]. In that study, chlorogenic acid, rutin, and p-coumaric acid were reported as the predominant compounds in pulp and seed extracts, particularly in aqueous extracts. In contrast, we detected chlorogenic acid only at low concentrations, whereas rosmarinic acid was among the most abundant phenolic compounds, especially in methanolic extracts. Rosmarinic acid has been previously associated with anticancer activity. For instance, Jim et al. [33] reported that this compound significantly reduced the viability of Hep-G2 hepatocarcinoma cells through multiple mechanisms, including induction of apoptotic cell death via upregulation of Bax, downregulation of Bcl-2, and activation of caspases 3 and 9. However, previous studies of *Randia* species have reported phenolic compounds, including flavonoids, coumarins, and phenolic acids. The seeds of species such as *R. monantha* contain the highest concentrations of polyphenols, with rutin, chlorogenic acid, and 4-coumaric acid among the most representative [16]. Coumarins, such as scopoletin; phenylpropanoid acids, such as chlorogenic acid; and phenolic acids, such as vanillic acid, have also been found. Compounds like phenylphosphonic acid have been found in the leaves of *R. aculeata* [19]. These differences may be attributed to several factors, including the plant organ analyzed (leaves and fruit vs. pulp and seeds) and the extraction solvents used. Solvent polarity strongly influences the extraction efficiency of phenolic compounds, with methanolic extracts often showing higher recovery of a broader range of phenolics. Additionally, both studies consistently detected rutin, supporting its relevance as a characteristic flavonoid in *Randia* spp.

These phytochemical differences were also reflected in the effects observed on HeLa cell viability. Although terpenoids were among the major classes of secondary metabolites identified in the extracts, particularly in the methanolic fruit extract, correlation analysis indicated that flavonoids showed the strongest association with reduced cell viability. Specifically, extracts with higher flavonoid content tended to show greater reductions in HeLa cell viability after 24 h of treatment, whereas no clear association was observed for terpenoids. Methanolic leaf extracts, which contained the highest flavonoid levels, produced one of the greatest reductions in HeLa cell viability. These exploratory correlations indicate an association between higher flavonoid content and lower HeLa cell viability at 24 h. However, given the small number of extracts analyzed (*n* = 6), these findings should not be interpreted as evidence that flavonoids are responsible for the observed biological effects. Because plant extracts are complex mixtures, synergistic interactions among metabolite classes are likely to contribute to the observed biological effects. The stronger effect observed in leaf extracts may be associated with the accumulation of defensive secondary metabolites in leaf tissues, which are directly exposed to environmental stress and herbivory. Such conditions can stimulate the biosynthesis of phytochemicals, including flavonoids and terpenoids, which are commonly involved in plant defense responses [34].

Recently, Martínez-Sánchez et al. [15] reported an IC_50_ of 25 µg/mL at 48 h for a hydroalcoholic fruit extract of *Randia* spp. in a colon adenocarcinoma cell line (CaCo-2). In the present study, the methanolic leaf extract showed an estimated IC_50_ of 4.87 µg/mL at 48 h in HeLa cells, indicating a greater effect on cell viability under the conditions evaluated. Similar antiproliferative activity has also been reported for *Randia*-derived silver nanoparticles. Bernabé-Antonio et al. [35] evaluated silver nanoparticles synthesized from a cell suspension culture of *Randia aculeata* against Hep3B, HepG2, A549, and HeLa cancer cell lines and reported IC_50_ values ≤ 7.6 µg/mL. These findings are particularly relevant because most previous biological studies and traditional uses of *Randia* spp. have focused almost exclusively on the fruit. In contrast, the present results suggest that leaf tissues remain comparatively underexplored despite their enriched phytochemical composition and marked effects on cancer cell viability. Therefore, these results highlight the potential of leaves as a promising source of bioactive compounds with antiproliferative properties.

The greater reduction in cell viability observed after 48 h indicates a time-dependent response to the extracts. Although mechanisms such as apoptosis, oxidative stress modulation, or cell-cycle alterations have been reported for plant-derived compounds, these mechanisms were not evaluated in the present study and therefore cannot be inferred from the MTT results alone. Similar time-dependent reductions in cell viability have been reported for several plant-derived extracts, with prolonged exposure enhancing cytotoxic activity in HeLa cells. For example, Gou et al. [36] demonstrated that an ursolic acid derivative induced time-dependent G0/G1 cell cycle arrest by downregulating cyclin D1 and CDK4 proteins in HeLa and MCF7 cells, and triggered endoplasmic reticulum stress and lysosomal dysfunction. Therefore, the greater activity observed after prolonged exposure to *Randia* spp. extracts may reflect progressive cellular alterations induced by terpenoids and other bioactive compounds in the extracts.

Compared with HeLa cells, DU-145 cells exhibited a distinct response across extraction solvents and plant tissues, suggesting differences in cellular sensitivity and mechanisms of action between the two cancer cell lines. Overall, HeLa cells showed greater reductions in cell viability in response to most *Randia monantha* extracts, particularly the methanolic and ethyl acetate leaf extracts. In contrast, the ethyl acetate fruit extract produced a marked reduction in DU-145 cell viability, especially at the highest concentration tested (350 µg/mL). These findings indicate that the effects of the extracts on cell viability varied between the two cell lines. However, the cellular mechanisms underlying these differences were not investigated in the present study. Furthermore, the exploratory correlation analysis in DU-145 cells showed different associations between metabolite classes and cell viability; given the limited number of extracts analyzed (*n* = 6), these trends should not be interpreted as evidence that specific compounds or metabolite classes are responsible for the observed effects.

These differences in biological response may also reflect the complex phytochemical composition of plant extracts. Plant extracts are naturally complex mixtures of flavonoids, phenolic compounds, terpenoids, and other secondary metabolites that may interact through complementary biological mechanisms [37,38,39]. Previous studies suggest that whole-plant preparations can exhibit greater biological activity than isolated compounds because of interactions among their phytochemical constituents. Secondary metabolites such as polyphenols and terpenoids may contribute through distinct yet complementary mechanisms [40]. For example, flavonoids have strong binding affinity for proteins and glycoproteins, whereas terpenoids show high affinity for biological membranes because of their lipophilic nature. These properties may enhance the overall biological activity of phytochemical mixtures when sufficient bioavailability is achieved. In cancer, these interactions may simultaneously modulate pathways associated with apoptosis, oxidative stress, inflammation, membrane permeability, and intracellular signaling, contributing to multi-target antiproliferative effects [37]. Therefore, the biological effects observed for *Randia monantha* extracts may reflect the complexity of their phytochemical composition; however, the contribution of individual compounds or interactions among metabolite classes cannot be established from the present data.

Although the present study demonstrates that *Randia monantha* extracts can reduce the viability of the evaluated cancer cell lines, the precise biological mechanisms underlying these effects remain unclear. Differences observed across cell lines, extraction solvents, and exposure times suggest that multiple cellular pathways may contribute to the biological response. Therefore, additional studies focused on apoptosis induction, cell cycle arrest, oxidative stress modulation, and migration-related pathways are necessary to better understand the mechanisms underlying the observed reductions in cell viability. Furthermore, the complex phytochemical composition of the extracts suggests that biological activity may not depend on a single compound but rather on interactions among multiple metabolites. In this context, future studies involving phytochemical fractionation, compound identification, and bioactivity-guided isolation will be important for determining the contributions of individual constituents and their potential interactions. Likewise, evaluating extract selectivity in non-cancerous cell lines will be essential to better assess their biological safety and therapeutic relevance. Complementary in vivo studies and bioavailability analyses will also be necessary to establish their pharmacological potential under physiological conditions.

## 4. Materials and Methods

### 4.1. Plant Material

The leaves and fruits used in this study were collected in Paso de Ovejas, Tolomé, Veracruz, Mexico (19°13.062′ N, 096°32.60′ W), with support from local producers. Morphometric characteristics of the plant were recorded, including phenological stage, canopy shape, branching type, bark texture, presence of lenticels, presence and number of spines at the apex, petiole characteristics, pubescence color, leaf arrangement, apex and base shape, adaxial and abaxial surface color, presence of pubescence, and type and color of leaf blade venation. Floral and fruit characteristics were also recorded, including flower and calyx color, peduncle length and diameter, and fruit shape, diameter, color, and number of fruits per inflorescence. Dr. Mireya Burgos-Hernández identified the species as *Randia monantha* Benth. at the CHAPA Herbarium (Herbario-Hortorio, Colegio de Postgraduados, Montecillo, Mexico). We deposited a voucher specimen in the CHAPA Herbarium under voucher number 159254.

The collected plant material was processed according to tissue type. The leaves were dehydrated in an oven at 40 °C (BLUE-M, Electronic Company/Blue Island, IL, USA) and subsequently ground to obtain a homogeneous powder. The fruits were processed fresh; the pulp was separated from the seeds and peel and homogenized using a blender prior to extraction. The processed plant materials were then weighed for subsequent extraction.

### 4.2. Extract Preparation

Crude extracts from *Randia monantha* were obtained through a sequential extraction with solvents of increasing polarity: hexane (Mc. Meyer^®,^ Mexico City, Mexico), ethyl acetate (Mc. Meyer^®^, Mexico City, Mexico), and methanol (Mc. Merck^®^, Sigma, Frankfurt, Germany). A total of 305.2 g of dried leaf material and 433.5 g of ground fruit pulp. The dried leaves were ground using a blender and standardized to a 2 mm particle size, whereas the fresh fruit pulp, previously separated from the seeds and peel, was homogenized using a blender.

The prepared plant materials were extracted using a 1:2 (g/mL) plant material-to-solvent ratio in 2 L glass containers. Sequential extraction was performed using pure hexane, pure ethyl acetate, and pure methanol, in that order. For each solvent, the plant material was macerated at 24 ± 2 °C for 24 h and subsequently sonicated for 10 min in static mode using a Branson 8510 ultrasonic bath (Branson Ultrasonics, Danbury, CT, USA), operating at a frequency of 40 kHz and a nominal power of 320 W. The extracts were then filtered through Whatman No. 1 filter paper. This procedure was repeated three times with each solvent before proceeding to the next solvent. The filtrates were concentrated using a rotary evaporator under reduced pressure generated by the vacuum pump. Although the rotary evaporator was not equipped with a pressure gauge, the vacuum generated by the pump provided the reduced-pressure conditions required for solvent evaporation.

### 4.3. Determination of Extraction Yield

The extraction yield (%) for each leaf and fruit extract was calculated as the ratio of the dry weight of the recovered crude extract to the initial dry weight of the corresponding plant material, multiplied by 100, using the following equation:

Extraction yield (%) = (dry weight of crude extract/dry weight of dry plant material) × 100.

### 4.4. Preliminary Phytochemical Screening

Qualitative phytochemical analyses were conducted to identify the principal classes of secondary metabolites in the *Randia manantha* extracts, including terpenes, flavonoids, phenolic compounds, alkaloids, saponins, and tannins.

Phenolic compounds were qualitatively detected using the Folin–Ciocalteu assay with modifications [41]. Briefly, 0.5 mL of each extract was mixed with 0.5 mL of 10% Folin–Ciocalteu reagent and 0.5 mL of 2.5% Na_2_CO_3_. Gallic acid (Sigma-Aldrich, St. Louis, MO, USA) was used as the positive control. The development of an intense blue coloration was considered indicative of a positive reaction for phenolic compounds.

Terpenoids were qualitatively detected using the Liebermann–Burchard test with modifications [42]. Briefly, 0.5 mL of each extract was evaporated to dryness in a water bath, and the resulting residue was redissolved in a few drops of chloroform. Subsequently, four drops of concentrated sulfuric acid (H_2_SO_4_) and four drops of acetic anhydride were added. Ursolic acid (Sigma-Aldrich, St. Louis, MO, USA) was used as the positive control.

Flavonoids were qualitatively detected using the Shinoda test with modifications [43]. Briefly, 0.5 mL of each extract was transferred into a screw-cap tube, followed by the addition of a magnesium ribbon and a few drops of concentrated hydrochloric acid (HCl). Quercetin extract was used as the positive control, while a tube containing only the reagents was used as the negative control. The development of a faint orange coloration in the effervescent solution was considered a positive reaction.

Alkaloids were qualitatively detected using the Dragendorff test with modifications [44]. Briefly, 0.5 mL of each extract was mixed with 2 mL of 1% HCl and heated in a water bath for 20 min. After cooling to room temperature, four drops of Dragendorff reagent were added. An alkaloid-containing extract of *Lupinus* spp. was used as the positive control, while methanol was used as the negative control. The development of an opaque orange coloration was considered indicative of a positive reaction.

Tannins were qualitatively detected using the ferric chloride test with modifications [45]. Briefly, 1 mL of each extract was mixed with 0.5 mL of distilled water and shaken, followed by the addition of a few drops of ferric chloride (FeCl_3_) solution. A standardized tannin extract was used as the positive control, while methanol was used as the negative control. The development of a blue-black or green coloration was considered indicative of a positive reaction.

Saponins were qualitatively detected using the foam test with modifications [45]. Briefly, 0.5 mL of each extract was mixed with 1 mL of distilled water and vigorously shaken for 1 min. The formation of a stable foam persisting for more than 1 min after shaking was considered indicative of a positive reaction. A 10 mg sample of zacate chi-chi suspended in 1 mL of distilled water was used as the positive control, while 1 mL of distilled water was used as the negative control.

The relative abundance of each metabolite group was estimated using a semiquantitative scale based on the intensity of the color reaction relative to the positive control, and groups were classified as intense (+++), moderate (++), weak (+), or absent (−).

#### 4.4.1. Thin-Layer Chromatography Detection

The presence of major secondary metabolite groups in the *Randia monantha* extracts was preliminarily evaluated by TLC on silica gel plates (10 × 10 cm). Mobile phase systems were selected according to the metabolite class analyzed, including ethyl acetate–methanol for phenolic compounds and tannins, dichloromethane–methanol–ammonium hydroxide for alkaloids, chloroform–methanol–water for flavonoids, chloroform–methanol for terpenoids, and ethyl acetate–formic acid–acetic acid–water for saponins.

Before development, the chromatographic chamber was saturated with the corresponding solvent system for approximately 30 min. Extract samples (10 µL) were applied to the silica plates in successive aliquots to improve spot visualization. Chromatographic separation was performed until the solvent front reached the appropriate migration distance.

After elution, metabolite groups were visualized using specific reagents for each compound class. The developed plates were then heated at 105–110 °C for approximately 5 min to enhance color development and facilitate compound detection.

#### 4.4.2. Identification of Secondary Metabolites by High-Performance Liquid Chromatography

The phytochemical profile of the *Randia monantha* extracts was evaluated by HPLC to identify flavonoids, phenolic acids, and terpenoids. For sample preparation, 20 mg of each extract was dissolved in HPLC-grade methanol (Sigma-Aldrich, USA) and filtered through 0.22 µm membrane filters (Millipore, Cork, Ireland) before chromatographic analysis.

We performed the analyses on an Agilent HPLC system (Agilent Technologies, Santa Clara, CA, USA) coupled to a photodiode array detector and equipped with a reversed-phase C18 column (250 × 4.6 mm, 5 µm). Compound separation was achieved using a gradient eluent consisting of water with 0.1% formic acid (solvent A) and acetonitrile (solvent B). The gradient program began at 10% B, increased to 60% within 30 min, then reached 90% at 40 min, was held for 5 min, and finally returned to the initial conditions.

Chromatographic runs were performed at a flow rate of 1.0 mL/min with an injection volume of 20 µL. Detection was monitored at 210, 254, 280, and 340 nm, based on the absorption characteristics of the metabolites analyzed. Compound assignments were performed by comparing the retention times and UV absorption spectra of peaks detected in the extracts with those of authentic reference standards analyzed under the same chromatographic conditions. Calibration curves were prepared using multiple concentrations of each reference standard, yielding linear responses with correlation coefficients (R^2^ ≥ 0.995).

Reference standards included the flavonoids rutin, quercetin, catechin, morin, hesperidin, phlorizin, naringenin, and phloretin, as well as the phenolic acids chlorogenic, caffeic, ferulic, gallic, and vanillic (Sigma-Aldrich, USA). Terpenoid compounds were identified by comparing retention times and UV absorption spectra with available analytical references and previously reported chromatographic data. Calibration curves were prepared using multiple concentrations of each standard, yielding linear responses with correlation coefficients (R^2^) ≥ 0.995.

Terpenoids were analyzed separately using a Zorbax Eclipse XDB C8 column (125 × 4.0 mm, 5 µm) (Agilent Technologies, Santa Clara, CA, USA) maintained at 40 °C. The mobile phase consisted of acetonitrile (80%) and water (20%), with a flow rate of 1.0 mL/min and an injection volume of 20 µL. Detection was performed at 215 and 220 nm. Terpenoid assignments were performed by comparing the retention times and UV absorption spectra of peaks detected in the extracts with those of authentic reference standards analyzed under the same chromatographic conditions. Reference standards corresponded to the terpenoids quantified in the extracts: carnosol, ursolic acid, stigmasterol, oleanolic acid, and β-sitosterol.

We evaluated method performance using standard validation parameters, including accuracy, precision, specificity, and sensitivity. The limits of detection (LOD) and quantification (LOQ) were estimated using signal-to-noise ratios of 3:1 and 10:1, respectively.

### 4.5. Cell Lines and Culture Conditions

The cell lines used to evaluate the activity of the extracts were HeLa (CCL-2) and DU-145 (CRL-HTB-81). The DU-145 (CRL-HTB-81) cell line was obtained from the American Type Culture Collection (ATCC). This cell line was isolated from a brain metastasis in a 69-year-old man with castration-resistant prostate cancer (CRPC). These cells express a mutated variant of the androgen receptor (T877A) and are therefore widely used to understand the progression of prostate cancer and drug resistance [46,47].

Meanwhile, HeLa cells are derived from cervical carcinoma in a 31-year-old woman. This cell line is distributed by ATCC and is considered the gold standard for CaCu, as it contains human papillomavirus 18 (HPV-18) sequences. In this study, the HeLa cell line was donated by Dr. Sara Elisa Herrera Rodríguez of CIATEJ.

HeLa cells were maintained in Dulbecco’s Modified Eagle Medium (DMEM) (Sigma-Aldrich, St. Louis, MO, USA; Cat. D6429) supplemented with 10% fetal bovine serum (FBS) (Biowest, Nuaillé, France; Cat. S1650-500) and 1% penicillin-streptomycin (Sigma-Aldrich, St. Louis, MO, USA; Cat. P4333). Meanwhile, DU-145 cells were maintained in Eagle’s Minimal Essential Medium (EMEM) (Sigma-Aldrich, St. Louis, MO, USA; Cat. M0643) supplemented with 10% FBS and 1% penicillin-streptomycin.

#### 4.5.1. Subculture and Passaging

Cells were cultured in P-60 Petri dishes and incubated at 37 °C in an incubator with 5% carbon dioxide and 95.5% relative humidity. We monitored cultures daily to rule out signs of contamination. In each assay, cells were allowed to reach approximately 80% confluence. Once this confluence was reached, the culture medium was replaced. Cells were washed with phosphate-buffered saline (PBS), and trypsin was added to facilitate detachment. The cells were incubated for 5 min and then observed under a microscope at ×10 magnification to confirm detachment. Subsequently, cells were manually counted using a Neubauer chamber under an optical microscope and subcultured into new P-60 Petri dishes containing fresh medium.

#### 4.5.2. Evaluation of Cell Viability by MTT Assay

The effects of the extracts on cell viability were evaluated using the MTT assay (3-(4,5-dimethylthiazol-2-yl)-2,5-diphenyltetrazolium bromide). The plant extract was dissolved in dimethyl sulfoxide (DMSO) and then diluted in culture medium to achieve final concentrations of 0 (control), 1, 10, 80, 200, and 350 µg/mL. We selected this concentration range based on preliminary exploratory assays to assess concentration-dependent effects on cell viability across a broad range.

We evaluated cell viability in HeLa cells at 24 h and 48 h. DU-145 cells were evaluated only at 48 h because preliminary assays indicated a more pronounced reduction in cell viability with prolonged exposure, enabling clearer comparisons among treatments. The final DMSO concentration across all treatments was kept below 0.2%, a level that did not affect cell viability, as confirmed by the vehicle control. DMSO-treated cells served as the negative control. HeLa and DU-145 cells were seeded in 96-well plates (5 × 10^3^ cells per well) and allowed to adhere for 24 h at 37 °C in a humidified atmosphere containing 5% CO_2_. After the adhesion period, the culture medium was replaced with fresh medium containing the corresponding extract concentrations (0, 1, 10, 80, 200, or 350 μg/mL), and the cells were incubated for the indicated treatment periods (24 or 48 h for HeLa; 48 h for DU-145). Subsequently, 7 μL of MTT solution was added to each well containing 200 μL of medium, and the plates were incubated for 4 h. The medium was then removed, 100 μL of isopropanol was added to dissolve the formazan crystals, and absorbance was measured at 595 nm. Cell viability (%) was calculated using the following equation:

Cell viability (%) = (Absorbance of treated cells/Absorbance of control cells) × 100.

### 4.6. Statistical Analysis

Data were expressed as mean ± standard deviation (SD) across three independent experiments. Statistical analyses were performed using GraphPad Prism 8.0.1 and R software (version 4.3.0), with significance evaluated at *p* < 0.05. We initially assessed differences among treatments using one-way analysis of variance (ANOVA), followed by Tukey’s post hoc test when the assumptions of normality and homogeneity of variances were met. For datasets that did not meet parametric assumptions, nonparametric analyses were performed using the Kruskal–Wallis test followed by Dunn’s multiple-comparison test with Bonferroni adjustment. Associations between metabolite content and cell viability were evaluated using Spearman’s rank correlation coefficient. We calculated correlations between the total contents of phenolics, flavonoids, and terpenoids in each extract and the percentage of HeLa cell viability at 350 µg/mL after 24 h and 48 h of exposure.

## 5. Conclusions

This study demonstrated that the phytochemical composition and effects of *Randia monantha* extracts on cancer cell viability varied according to both extraction solvent and plant tissue. Among the evaluated extracts, the methanolic leaf extract showed the highest phenolic and flavonoid contents and the greatest reduction in HeLa cell viability, with estimated IC_50_ values of approximately 25 µg/mL at 24 h and 4.87 µg/mL at 48 h. The ethyl acetate leaf extract also markedly reduced HeLa cell viability after 48 h of exposure, with an estimated IC_50_ of approximately 14 µg/mL. In contrast, DU-145 cells showed a different response pattern, with the ethyl acetate fruit extract exhibiting the clearest concentration-dependent reduction in cell viability and an estimated IC_50_ of 159 µg/mL at 48 h.

Based on these findings, the methanolic leaf extract should be prioritized for further investigation in the HeLa model, whereas the ethyl acetate fruit extract represents the most suitable candidate for further evaluation in the DU-145 model. Future studies should employ denser concentration–response designs around the estimated IC_50_ values to obtain more robust potency estimates. In particular, the marked effect of the methanolic leaf extract at relatively low concentrations supports its prioritization for phytochemical fractionation and bioactivity-guided studies aimed at identifying the constituents associated with the observed biological activity.

Nevertheless, these findings should be considered preliminary because the biological effects were evaluated using the MTT assay alone and without a non-cancerous cell control. Therefore, the observed reductions in cell viability cannot by themselves establish inhibition of cell proliferation, define the underlying mechanisms, or demonstrate selectivity toward cancer cells. Further studies should confirm these effects using complementary viability or proliferation assays and evaluate selectivity in appropriate non-cancerous cell models. Subsequent bioactivity-guided fractionation and characterization of the active constituents would provide a stronger basis for determining whether the observed in vitro activity warrants further pharmacological and in vivo investigation.

## Figures and Tables

**Table 1:** Preliminary phytochemical screening of *Randia monantha* extracts.

Metabolites	LH	LEA	LM	FH	FEA	FM
Phenols	+	+	++	+	+	++
Alkaloids	−	+	+	−	−	+
Tannins	−	+	−	−	−	−
Flavonoids	−	−	−	−	−	+
Terpenoids	++	+++	+	+	+++	+++
Saponins	+	++	−	−	−	+++

LH: Leaf hexane extract; LEA: Leaf ethyl acetate extract; LM: Leaf methanolic extract; FH: Fruit hexane extract; FEA: Fruit ethyl acetate extract; FM: Fruit methanolic extract. Intense presence (+++), moderate presence (++), weak presence (+), and absence of compound (−).

**Table 2:** TLC of *Randia monantha* extracts.

Metabolite Class	Reference Standard	Rf Standard	Rf Observed in Extracts
Alkaloids	*Lupinus* sp. extract	0.73	0.94–1.00
Phenolics	Gallic acid	0.76	0.76–0.97
Flavonoids	Quercetin	0.97	0.89–0.97
Tannins	Tannic acid	0.90	0.92–0.99
Saponins	Soy saponin	0.06	0.51–1.00
Terpenoids	Ursolic acid	0.04	0.04–0.95

**Table 3:** Phenols identified and quantified by HPLC in *Randia monantha* extracts.

Compound	LH	FH	LEA	FEA	LM	FM
	**(mg metabolite/g extract)**					
Sinapic acid	ND	ND	ND	ND	ND	0.06 ± 0.06
p-Hydroxybenzoic acid	ND	ND	ND	0.52 ± 0.12	0.75 ± 0.014	ND
β-Resorcylic acid	0.47 ± 0.001	ND	ND	ND	ND	ND
Syringic acid	ND	ND	ND	ND	ND	ND
Chlorogenic acid	ND	ND	ND	ND	ND	0.06 ± 0.02
Ferulic acid	ND	ND	ND	0.42 ± 0.78	ND	0.15 ± 0.03
Rosmarinic acid	0.34 ± 0.02	0.429 ± 0.009	ND	ND	5.81616 ± 0.142	2.52 ± 0.72
Protocatechuic acid	ND	0.306 ± 0.0004	ND	0.949 ± 0.145	0.873 ± 0.001	0.49 ± 0.06
Vanillic acid	ND	ND	ND	1.16 ± 0.25	ND	0.11 ± 0.05
TOTAL	0.81	0.73	ND	3.06	7.44	3.62

Data are expressed as mg of metabolite per g of dry extract (mg g^−1^ extract) and presented as mean ± SD (*n* = 3). LH: Leaf hexane extract; FH: Fruit hexane extract; LEA: Leaf ethyl acetate extract; FEA: Fruit ethyl acetate extract; LM: Leaf methanolic extract; FM: Fruit methanolic extract. ND: not detected.

**Table 4:** Flavonoids identified and quantified by HPLC in *Randia monantha*. extracts.

Compound	LH	FH	LEA	FEA	LM	FM
	**(mg metabolite/g extract)**					
Kaempferol	ND	ND	0.40 ± 0.007	ND	0.23 ± 0.007	ND
Isorhamnetin	ND	ND	0.16 ± 0.005	ND	ND	0.06 ± 0.10
Apigenin	ND	0.11 ± 0.09	ND	0.202 ± 0.005	ND	0.21 ± 0.007
Rutin	0.86 ± 0.05	0.45 ± 0.26	0.53 ± 0.02	0.228 ± 0.132	5.46 ± 0.07	ND
Morin	0.29 ± 0.25	ND	ND	ND	ND	ND
Catechin	0.26 ± 0.15	0.13 ± 0.07	ND	ND	1.02 ± 0.09	0.25 ± 0.07
Hesperidin	ND	ND	ND	ND	0.67 ± 0.03	0.27 ± 0.06
Phlorizin	ND	ND	ND	0.65 ± 0.13	0	0.18 ± 0.04
Naringenin	ND	ND	ND	ND	0.23 ± 0.08	ND
Phloretin	ND	ND	ND	ND	0.21 ± 0.11	ND
TOTAL	1.43	0.70	1.09	1.08	7.84	0.99

Data are expressed as mg of metabolite per g of dry extract (mg g^−1^ extract) and presented as mean ± SD (*n* = 3). LH: Leaf hexane extract; FH: Fruit hexane extract; LEA: Leaf ethyl acetate extract; FEA: Fruit ethyl acetate extract; LM: Leaf methanolic extract; FM: Fruit methanolic extract. ND: not detected.

**Table 5:** Terpenes identified and quantified by HPLC in extracts of *Randia monantha*. Data are expressed as mean ± standard deviation (SD) of three replicate measurements (n = 3).

Compound	LH	FH	LEA	FEA	LM	FM
	**(mg metabolite/g extract)**					
Carnosol	ND	ND	0.06 ± 0.008	ND	0.0006 ± 0.001	ND
Ursolic acid	0.79 ± 0.11	ND	3.24 ± 0.29	ND	0.42 ± 0.33	ND
Stigmasterol	32.4 ± 1.22	51.93 ± 1.01	74.87 ± 1.38	72.26 ± 0.14	8.08 ± 0.86	45.63 ± 0.47
Oleanolic acid	18.58 ± 0.12	1.17 ± 0.90	110.42 ± 1.14	2.14 ± 1.8	22.97 ± 1.52	0.98 ± 0.83
β-sitosterol	20.53 ± 1.20	4.30 ± 1.12	ND	ND	ND	156.52 ± 2.44
TOTAL	72.30	57.41	188.61	74.4	31.48	203.14

LH: Leaf hexane extract; FH: Fruit hexane extract; LEA: Leaf ethyl acetate extract; FEA: Fruit ethyl acetate extract; LM: Leaf methanolic extract; FM: Fruit methanolic extract. ND: not detected.

**Table 6:** Spearman correlation coefficients (ρ) between total secondary metabolite contents (terpenoids, flavonoids, and phenolic compounds) and cell viability in HeLa and DU-145 cells. Correlations were calculated using viability values obtained at 350 µg/mL, the concentration at which the greatest reductions in cell viability were observed. Values in parentheses correspond to *p*-values. The sample size was *n* = 6 extracts for all analyses.

Metabolite Class	HeLa 24 h (*p*)	HeLa 48 h (*p*)	DU-145 48 h (*p*)
Terpenoids	0.20 (0.70)	0.20 (0.704)	−0.60 (0.20)
Flavonoids	−0.83 (0.042)	−0.66 (0.15)	0.77 (0.07)
Phenols	−0.25 (0.62)	0.20 (0.70)	−0.14 (0.78)

## Data Availability

The data supporting the findings of this study are available from the corresponding author upon reasonable request.

## Supplementary Materials

The following supporting information can be downloaded at: https://www.mdpi.com/article/10.3390/molecules31173095/s1, Table S1: Phenolic acids and flavonoids detected in methanolic leaf and fruit extracts of *Randia monantha* by HPLC analysis; Table S2: Terpenoid compounds identified in methanolic leaf and fruit extracts of *Randia* spp. using HPLC analysis.

## Abbreviations

ANOVA	Analysis of Variance
DMEM	Dulbecco’s Modified Eagle Medium
EMEM	Eagle’s Minimum Essential Medium
DU-145	Human prostate cancer cell line
FEA	Fruit ethyl acetate extract
FH	Fruit hexane extract
FM	Fruit methanolic extract
HPLC	High-Performance Liquid Chromatography
HeLa	Human cervical cancer cell line
IC_50_	Half maximal inhibitory concentration
LEA	Leaf ethyl acetate extract
LH	Leaf hexane extract
LM	Leaf methanolic extract
MTT	3-(4,5-dimethylthiazol-2-yl)-2,5-diphenyltetrazolium bromide
ND	Not detected
Rf	Retention factor
SD	Standard deviation
TLC	Thin-layer chromatography
