# Correction: Delgado-Tiburcio et al. Phytochemical Characterization and Effects of *Randia monantha* Benth. Leaf and Fruit Extracts on the Viability of Human Cancer Cell Lines. *Molecules* 2026, *31*, 3095

**DOI:** 10.3390/molecules31193396

**Published:** 2026-09-24

**Authors:** Eugenia Elisa Delgado-Tiburcio, Ivonne Pérez-Bautista, Ramón Marcos Soto-Hernández, Lucero del Mar Ruiz-Posadas, Guadalupe Elizabeth Jimenez-Gutierrez, Axelle Renodon-Cornière, Sara Elisa Herrera-Rodríguez, Ahtziri Socorro Carranza-Aranda, Jonathan J. Magaña, José M. Padrón, Luz A. Guerrero-Lagunes

**Affiliations:** 1 Botany Department, Colegio de Postgraduados, Campus Montecillo, km 36.5 Carretera México-Texcoco, Texcoco 56230, Mexico; delgado.eugenia@colpos.mx (E.E.D.-T.); perez.ivonne@colpos.mx (I.P.-B.); guerrero.luz@colpos.mx (L.A.G.-L.); 2 Genomic Medicine Laboratory, Instituto Nacional de Rehabilitación Luis Guillermo Ibarra Ibarra, Mexico City 14389, Mexico; gjimenezg@cinvestav.mx (G.E.J.-G.); magana.jj@tec.mx (J.J.M.); 3 Nantes Université, Centre National de la Recherche Scientifique (CNRS), Unité en Sciences Biologiques et Biotechnologies (US2B), UMR 6286, F-44000 Nantes, France; axelle.renodon-corniere@univ-nantes.fr; 4 Southeast Unit, Centro de Investigación y Asistencia en Tecnología y Diseño del Estado de Jalisco (CIATEJ), Carretera km 5.5, Sierra Papacal–Chuburná, Chuburná, Merida 97302, Mexico; sherrera@ciatej.mx; 5 Department of Philosophical, Methodological, and Instrumental Disciplines, University Center for Health Sciences (CUCS), University of Guadalajara, Guadalajara 44340, Mexico; ahtziricarranza19@gmail.com; 6 Department of Bioengineering, School of Engineering and Sciences, Tecnologico de Monterrey, Mexico City Campus, Mexico City 14380, Mexico; 7 BioLab, Instituto Universitario de Bio-Orgánica “Antonio González” (IUBO-AG), Universidad de La Laguna, Astrofísico Francisco Sánchez 2, E-38206 La Laguna, Spain; jmpadron@ull.es

**Error in Figure**

In the original publication [1], there was a mistake in Figures 1–6 as published. Incorrect figure images were included in the published version. The corrected Figure 1, Figure 2, Figure 3, Figure 4, Figure 5 and Figure 6 appear below.

**Additional Affiliation**

In the published publication, there was an error regarding the affiliation for Axelle Renodon-Cornière. The updated affiliation 3 should be: Nantes Université, Centre National de la Recherche Scientifique (CNRS), Unité en Sciences Biologiques et Biotechnologies (US2B), UMR 6286, F-44000 Nantes, France; axelle.renodon-corniere@univ-nantes.fr. 

The authors state that the scientific conclusions are unaffected. This correction was approved by the Academic Editor. The original publication has also been updated.

## Figures and Tables

**Figure 1:**
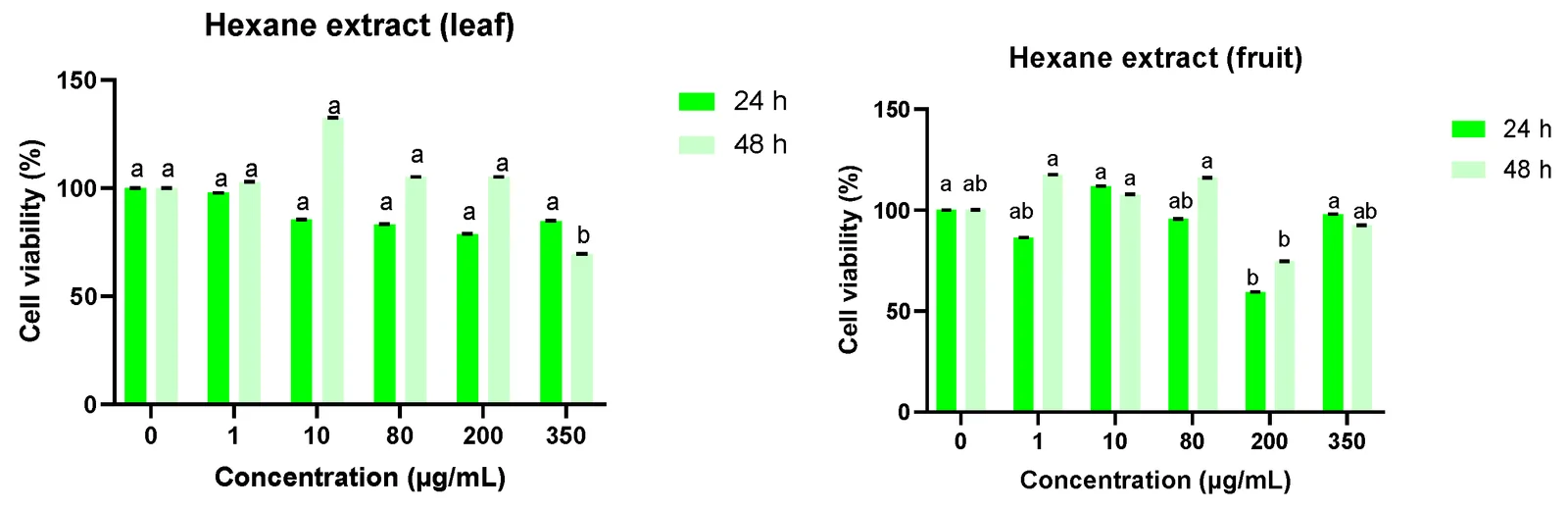
Effect of hexane extracts of *Randia monantha* on HeLa cell viability after 24 h and 48 h of treatment. Cell viability (%) was calculated relative to the untreated cells (C). Error bars represent standard deviation (SD, *n* = 3). Different letters indicate significant differences among treatments according to Tukey’s post hoc test (*p* < 0.05).

**Figure 2:**
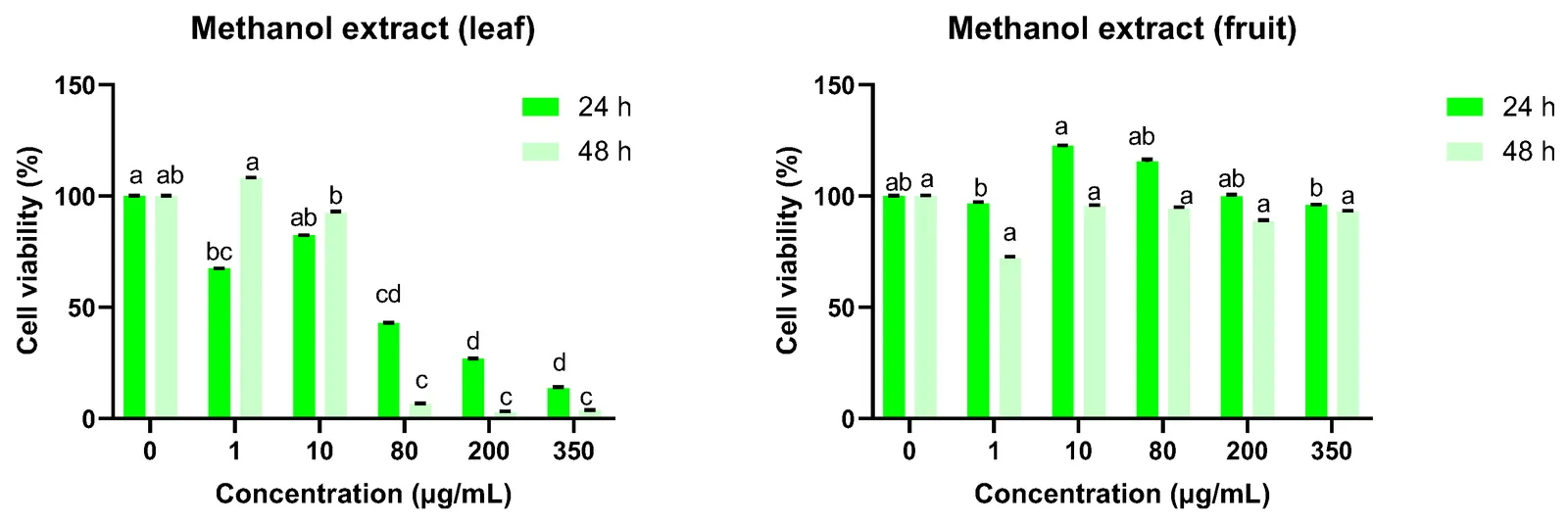
Effect of methanolic extracts of *Randia monantha* on HeLa cell viability after 24 h and 48 h of treatment. Cell viability (%) was calculated relative to the untreated cells (C). Data are presented as mean ± standard deviation (SD) of three independent experiments (*n* = 3). Different letters indicate significant differences among treatments according to Tukey’s post hoc test (*p* < 0.05).

**Figure 3:**
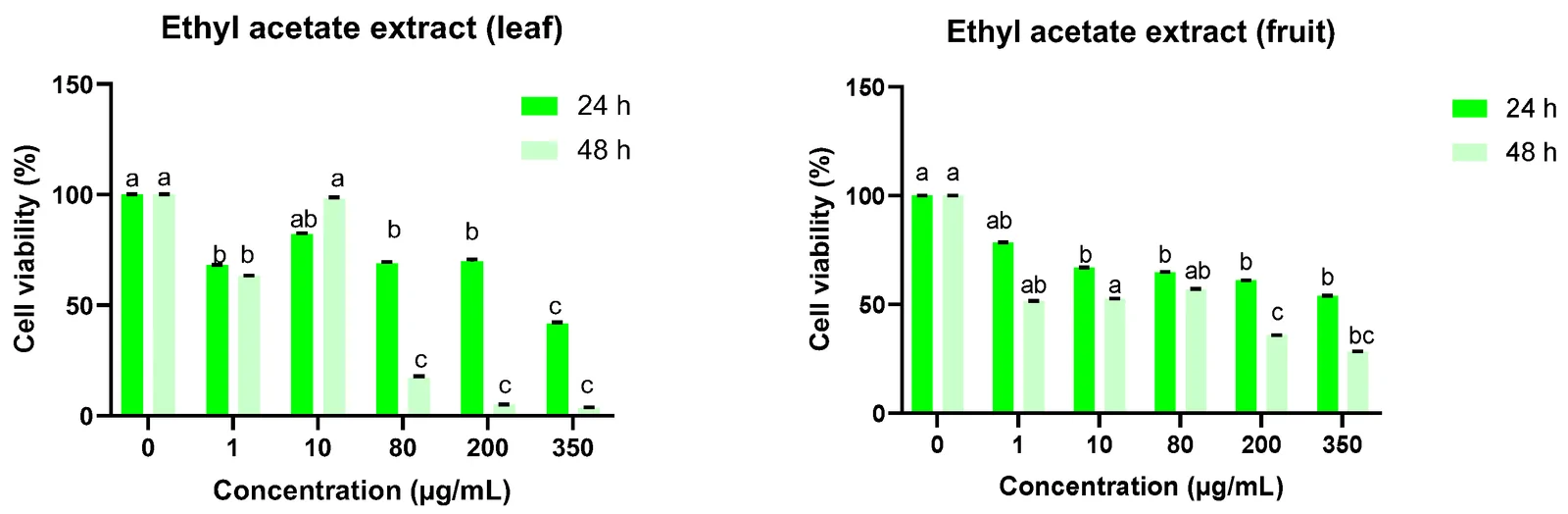
Effect of ethyl acetate extracts of *Randia monantha* on HeLa cell viability after 24 h and 48 h of treatment. Cell viability (%) was calculated relative to the untreated cells (C). Data are presented as mean ± standard deviation (SD) of three independent experiments (*n* = 3). Different letters indicate significant differences among treatments according to Tukey’s post hoc test (*p* < 0.05).

**Figure 4:**
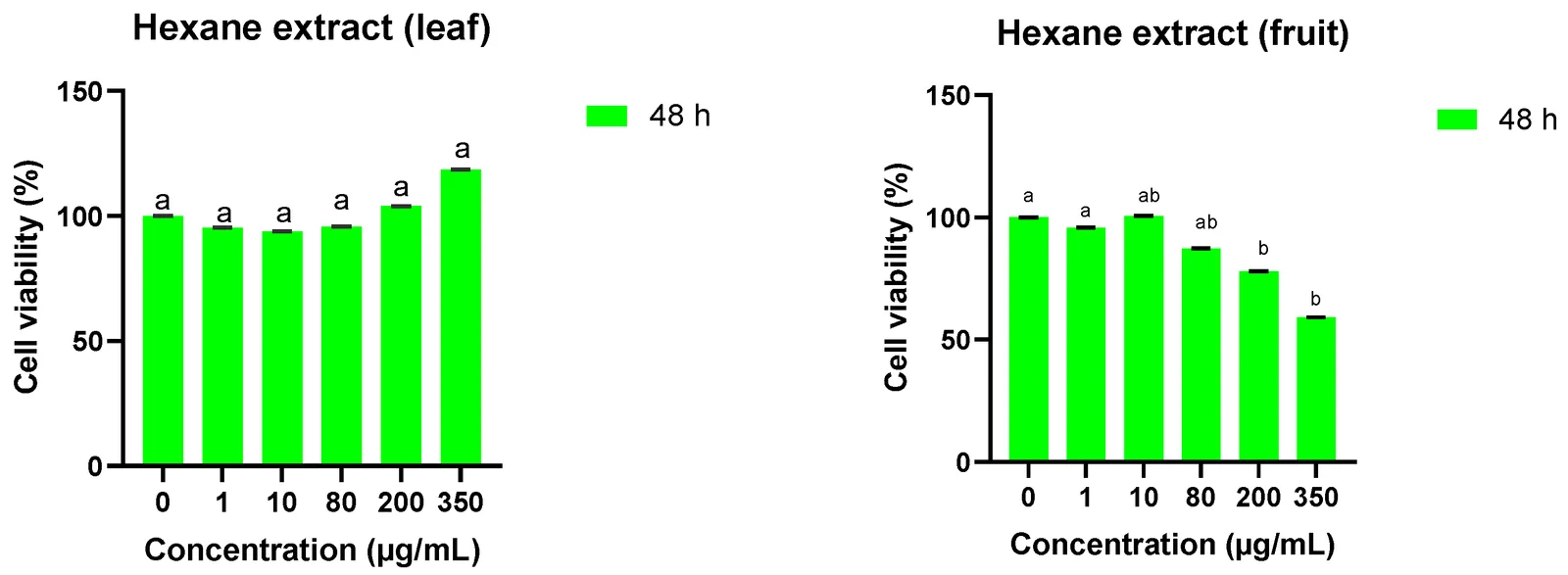
Effect of hexane extracts of *Randia monantha* on DU-145 cell viability after 48 h of treatment. Cell viability (%) was calculated relative to the untreated cells (C). Data are presented as mean ± standard deviation (SD) of three independent experiments (*n* = 3). Different letters indicate significant differences among treatments according to Tukey’s post hoc test (*p* < 0.05).

**Figure 5:**
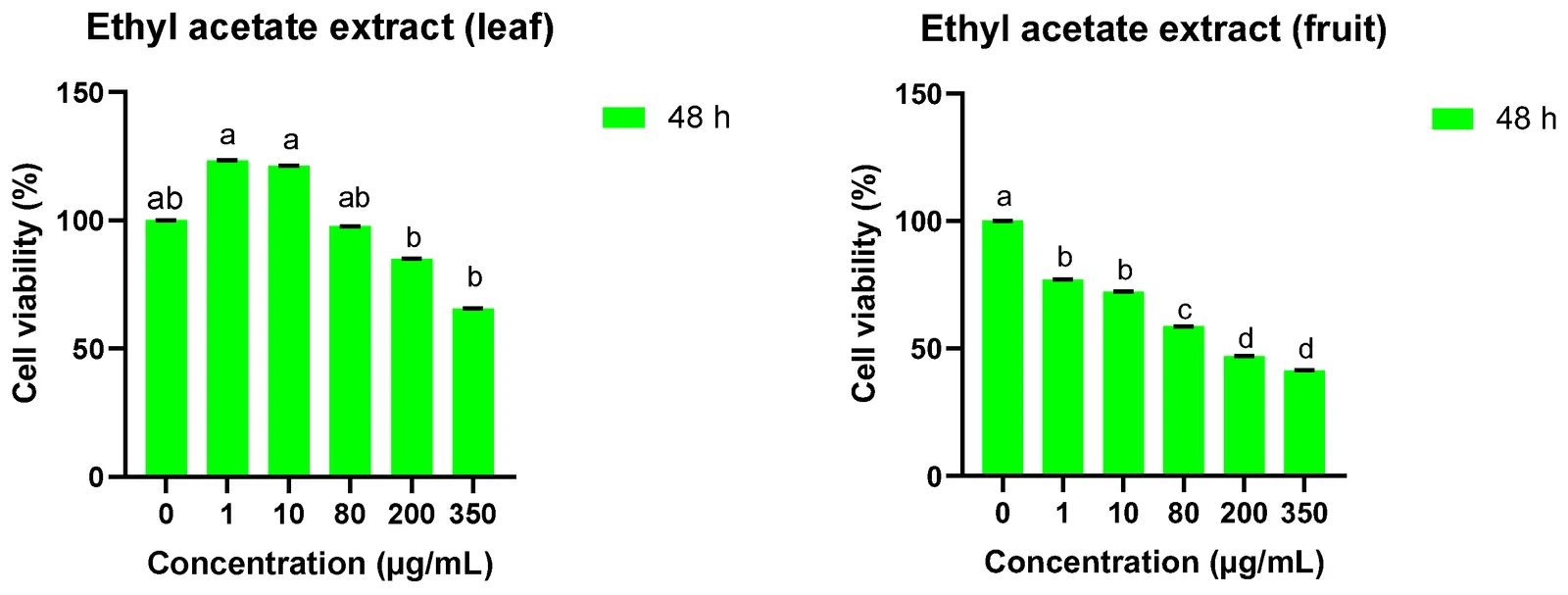
Effect of ethyl acetate extracts of *Randia monantha* on DU-145 cell viability after 48 h of treatment. Cell viability (%) was calculated relative to the untreated control cells (C). Data are presented as mean ± standard deviation (SD) of three independent experiments (*n* = 3). Different letters indicate significant differences among treatments according to Tukey’s post hoc test (*p* < 0.05).

**Figure 6:**
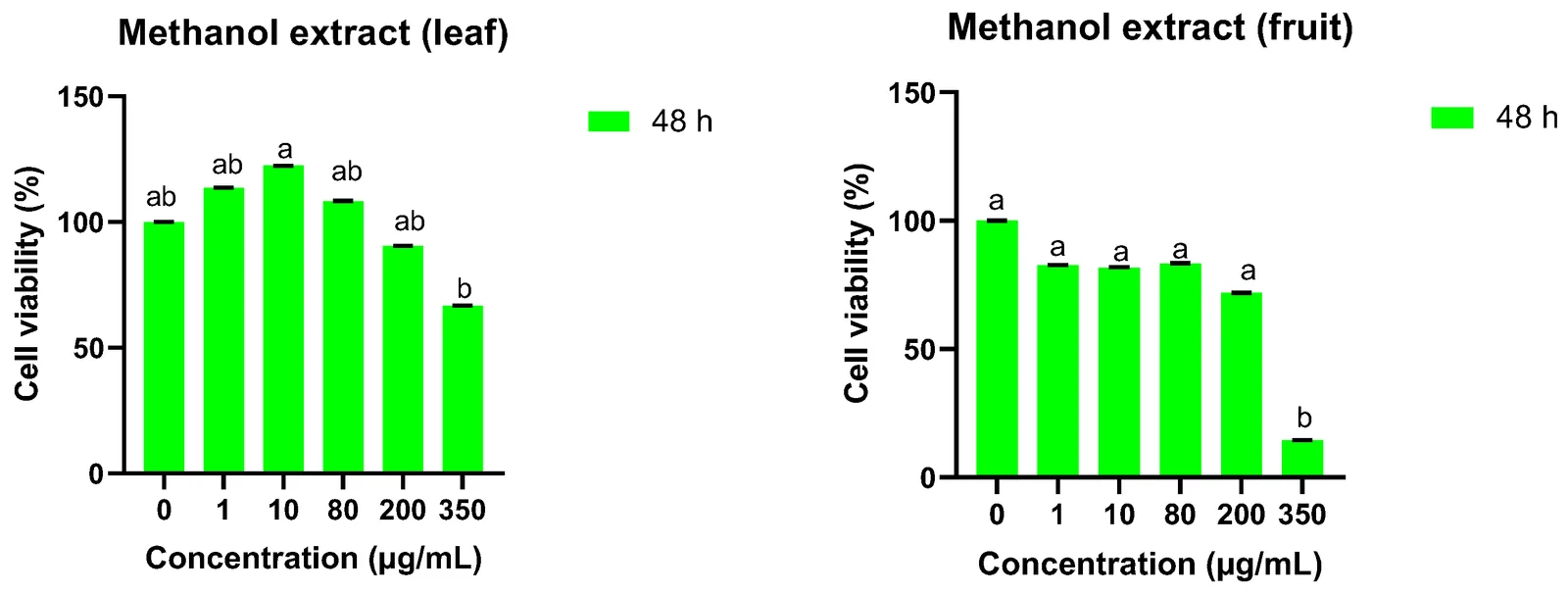
Effect of methanol extracts of *Randia monantha* on DU-145 cell viability after 48 h of treatment. Cell viability (%) was calculated relative to the untreated control cells (C). Data are presented as mean ± standard deviation (SD) of three independent experiments (*n* = 3). Different letters indicate significant differences among treatments according to Tukey’s post hoc test (*p* < 0.05).

## References

[1] Delgado-Tiburcio E.E., Pérez-Bautista I., Soto-Hernández R.M., Ruiz-Posadas L.d.M., Jimenez-Gutierrez G.E., Renodon-Cornière A., Herrera-Rodríguez S.E., Carranza-Aranda A.S., Magaña J.J., Padrón J.M. (2026). Phytochemical Characterization and Effects of *Randia monantha* Benth. Leaf and Fruit Extracts on the Viability of Human Cancer Cell Lines. Molecules.

