# Supplementary material for: Phytochemical Characterization and Effects of Randia monantha Benth. Leaf and Fruit Extracts on the Viability of Human Cancer Cell Lines
Source: Molecules. 2026 Sep 3;31(17):3095. doi: 10.3390/molecules31173095 (PMC13567197; doi:10.3390/molecules31173095)
Supplement: Supplementary file 1 [file molecules-31-03095-s001.zip › molecules-4511075-supplementary.pdf]

Compound	Rt (min)	Peak area	Relative abundance	Extract
Kaempferol	17.502	31.2539	Low	LM
Rutin	5.569	1463.67	High	LM
Catechin	2.565	96.123	Low	LM
Hesperidin	8.156	60.1708	Low	LM
Naringenin	15.813	13.3701	Low	LM
Phloretin	16.324	58.89	Low	LM
Gentisic acid	3.572	1126.98	High	LM
p-Hydroxybenzoic acid	2.350	2046	High	LM
Rosmarinic acid	8.740	732.161	High	LM
Protocatechuic acid	2.100	887.257	High	LM
Gentisic acid	3.336	298.85	Moderate	FM
Sinapic acid	6.832	176.97	Moderate	FM
Chlorogenic acid	3.602	33.717	Low	FM
Ferulic acid	5.685	145.637	Moderate	FM
Rosmarinic acid	8.695	353.513	Moderate	FM
Vanillic acid	3.300	206.354	Moderate	FM
Gallic acid	1.030	1275.85	High	FM
Apigenin	17.095	34.94	Low	FM
p-Coumaric acid	4.950	78.337	Low	FM
Catechin	2.588	26.3029	Low	FM
Hesperidin	7.821	27.2326	Low	FM
Phlorizin	9.103	87.78	Low	FM

Supplementary Table S1. Phenolic acids and flavonoids detected in methanolic leaf and fruit extracts of *Randia monantha* by HPLC analysis.

Compound	Rt (min)	Peak area	Relative abundance	Extract
Carnosol	2.576	129.37	Moderate	LM
Ursolic acid	2.968	21.816	Low	LM
Stigmasterol	3.480	73.4328	Low	LM
Oleanolic acid	4.686	919.812	High	LM
Carnosol	2.586	468.75	Moderate	FM
Stigmasterol	3.153	74.93	Low	FM
Oleanolic acid	4.640	31.662	Low	FM
β-sitosterol	20.528	ND	ND	FM

Supplementary Table S2. Terpenoid compounds identified in methanolic leaf and fruit extracts of *Randia* spp. using HPLC analysis.
